# Preparation and evaluation of *Brucella* T4SS recombinant proteins in serodiagnosis of human brucellosis based on TMT-based proteomics technology

**DOI:** 10.3389/fcimb.2024.1514046

**Published:** 2025-01-16

**Authors:** Qi Wu, Chen Sun, Liping Guo, Yujia Xie, Jinpeng Zhang, Dehui Yin

**Affiliations:** ^1^ Jiangsu Engineering Research Center of Biological Data Mining and Healthcare Transformation, Xuzhou Medical University, Xuzhou, Jiangsu, China; ^2^ Department of Clinical Laboratory, Huai’an Hospital of Huai’an City, Huai’an, China; ^3^ Key Laboratory of Human Genetics and Environmental Medicine, Xuzhou Medical University, Xuzhou, China

**Keywords:** brucellosis, type IV secretion system, VirB proteins, TMT proteomics, serodiagnosis

## Abstract

**Introduction:**

Brucellosis, a significant zoonotic infectious disease, poses a global health threat. Accurate and efficient diagnosis is crucial for prevention, control, and treatment of brucellosis. VirB proteins, components of the Type IV secretion system (T4SS) in *Brucella*, play a pivotal role in bacterial virulence and pathogenesis but have been understudied for their diagnostic potential.

**Methods:**

Tandem Mass Tag (TMT) proteomics technology was utilized to identify highly expressed VirB proteins from wild-type *Brucella* strains. Recombinant T4SS proteins were prepared, and an indirect ELISA method was established for serological diagnosis of human brucellosis.

**Results:**

Seven T4SS proteins (rVirB3, rVirB4, rVirB9, rBMEII0036, rVirB8, rVirB11, and rVirB10) were expressed used to construct the indirect ELISA method which showed high diagnostic accuracy. Sensitivity and specificity of the proteins exceeded 0.9100 and 0.9167, respectively, demonstrating good performance comparable to traditional LPS and Rose Bengal Ag antigens. Cross-reactivity was observed in a limited number of serum samples from febrile patients without brucellosis.

**Conclusions:**

The study highlights the potential of VirB proteins as novel diagnostic antigens for human brucellosis. Future research can further optimize the use of VirB proteins in diagnostic assays and explore their applications in vaccine development.

## Introduction

Brucellosis, a severe zoonotic infectious disease caused by bacteria of the genus *Brucella*, poses a significant global public health threat due to its widespread prevalence (Pappas et al., 2006). This disease not only hampers the development of the livestock industry but also spreads to humans through direct contact with infected animals, ingestion of inadequately processed dairy products, or inhalation of aerosols containing the bacteria (Fuchs et al., 2016; Abdel-Hamid et al., 2021). Clinical symptoms in humans include fever, fatigue, and joint pain, and in severe cases, it can lead to complications such as endocarditis, arthritis, and even death (Pappas et al., 2005; Franco et al., 2007). Therefore, the development of efficient and accurate diagnostic methods for brucellosis is crucial for its prevention, control, and treatment.

Currently, the diagnosis of brucellosis mainly relies on various methods, including pathogen detection, serological testing, and molecular biology techniques. Among these, serological testing is widely used due to its simplicity, low cost, and ability to reflect the immune status of the patient to some extent (Araj, 2010; Yagupsky et al., 2019). However, most existing serological diagnostic antigens are based on the cell wall components or outer membrane proteins of *Brucella*. Although these antigens possess certain immunogenicity, their diagnostic sensitivity and specificity are often compromised in practical applications due to factors such as the patient’s immune status, the stage of infection, and cross-reactivity (Chart et al., 1992; Bonfini et al., 2018).

VirB proteins, as key components of the Type IV secretion system (T4SS) in *Brucella*, play an important role in the interaction between the bacteria and host cells (Boschiroli et al., 2002; Zheng et al., 2024). The VirB system is not only involved in the survival and replication of *Brucella* within host cells but is also closely related to its pathogenic mechanisms. Despite the crucial role of VirB proteins in the biological functions of *Brucella*, there has been little research systematically analyzing their value in the serological diagnosis of human brucellosis. This gap in research limits our understanding of the pathogenic mechanisms of brucellosis and hinders the development and application of novel diagnostic antigens.

In this study, we aim to utilize Tandem Mass Tag (TMT) proteomics technology to prepare and evaluate the value of *Brucella* VirB proteins in the serological diagnosis of human brucellosis. TMT technology, known for its high sensitivity and high throughput in protein quantification, allowed for precise identification and quantification of proteins in complex biological samples, providing strong technical support for the screening and validation of novel diagnostic antigens (Moulder et al., 2018; Deng et al., 2022). Through TMT proteomics, we identified highly expressed VirB proteins in wild-type *Brucella* strains, prepared recombinant Type IV secretion system proteins, and established a serological detection method based on these proteins. The goal was to enhance the sensitivity and specificity of brucellosis diagnosis, thereby offering new antigen choices for the early diagnosis and prevention of the disease.

## Materials and methods

### Serum samples and bacterial strains

In this study, a total of 100 positive and 96 negative serum samples were obtained from the Xuzhou Center for Disease Control and Prevention, all confirmed as positive or negative through tube agglutination tests. Additionally, serum from 40 febrile patients infected with other pathogens (stored in the laboratory, with detailed information available in **Supplementary File 1**: Cross-Reactivity Assessment) was used to evaluate the cross-reactivity of the developed method. To identify highly expressed proteins in wild strain of *Brucella abortus*, as well as to discover antigenic proteins that can be utilized in the diagnosis of human brucellosis, the vaccine strain *Brucella abortus* A19 and the wild-type *Brucella abortus* DT21, both isolated and preserved by the China Animal Health and Epidemiology Center, were also utilized in this study.

### Proteomics analysis

#### Bacterial culture

The preserved bacterial strain was inoculated into 500 mL of Tryptic Soy Broth (TSB, STBMTSB12, Millipore, USA) medium and incubated at 37°C with shaking for 24-48 hours. After incubation, 5 mL of 1% formaldehyde was added to inactivate the bacteria, which was then stored at 4°C for later use.

#### Proteomics analysis

Proteomics analysis was performed according to standard protocols referenced from the literature, including steps such as protein extraction and quantification, protein digestion and TMT labeling, LC-MS/MS analyses, and qualitative and quantitative analysis of proteins (Deng et al., 2022). The peak intensities of TMT-tagged reagent ions were quantitatively compared across samples (**Supplementary Table S1**). The statistical analysis of the differential proteins identified was conducted using analysis of variance (ANOVA), with a significance threshold set at p < 0.05. Proteins exhibiting a fold change greater than 1.2 (ratio≥1.2) or less than 0.83(ratio ≤ 0.83) were classified as highly expressed proteins.

### Preparation of recombinant T4SS proteins

Based on TMT proteomics analysis, we selected highly expressed T4SS proteins from the wild-type strain. The amino acid sequences of these proteins were retrieved from the NCBI protein database. Using the UniProt website (https://www.uniprot.org/uniprotkb), we predicted and removed transmembrane regions, signal peptides, and hydrophobic regions, constructing the recombinant sequences of the Type IV secretion system proteins. The sequence was then submitted to Beijing Protein Innovation Co., LTD. for codon optimization to suit prokaryotic expression. Gene synthesis was carried out, and a 6xHis tag was added to facilitate subsequent protein purification.

The synthesized recombinant protein gene was cloned into the pET30a expression vector. The vector was then transformed into competent BL21 cells for IPTG-induced expression. The procedure was conducted as follows: Competent BL21 cells, previously stored at -80°C, were thawed on ice and subsequently mixed with pET30a(+). The mixture was incubated on ice for 30 minutes, followed by a heat shock at 42°C for 90 seconds, after which the cells were immediately cooled on ice for 2 minutes. Subsequently, 800 μL of LB medium (L113084, Aladdin, USA) was added, and the cells were incubated at 37°C for 45 minutes. The culture was then centrifuged at 3214×g (Eppendorf Centrifuge 5810R, Germany) for 3 minutes, with most of the supernatant discarded, leaving approximately 100-150 μL, in which the cells were resuspended. The resuspended cells were plated onto LB agar plates containing the appropriate antibiotic and incubated overnight at 37°C. The following day, the cultured bacterial solution was transferred into 250 mL of LB liquid medium supplemented with the corresponding antibiotic and incubated at 37°C with shaking at 200 rpm using DHZ-DA Large-capacity full-temperature oscillator (Changzhou Guoyu Instrument Manufacturing Co., China) until the optical density at 600 nm (OD600) reached 0.6-0.8. Induction of protein expression was achieved by adding 0.5 mM IPTG (16758, Sigma, Germany) and continuing the incubation at 37°C for 4 hours. The culture was then centrifuged at 8228×g for 6 minutes, the supernatant was discarded, and the cell pellet was collected. The pellet was resuspended in 20-30 mL of 10 mM Tris-HCl (pH 8.0) solution and subjected to ultrasonic disruption (500 W, 180 cycles, 5 seconds per cycle with 5-second intervals). A 100 μL aliquot of the disrupted bacterial suspension was centrifuged at 18514×g for 10 minutes. Of the resulting supernatant, 50 μL was transferred to a separate Eppendorf tube, while the pellet was resuspended in 50 μL of 10 mM Tris-HCl (pH 8.0) solution. To ascertain the presence of the target protein in either the supernatant or the pellet, 12% SDS-PAGE (P0012AC, Beyotime Biotechnology, Shanghai, China) electrophoresis was performed for subsequent purification.

The nickel column (Ni Sepharose 6 Fast Flow, GE Healthcare) was washed with deionized water until the pH reached 7.0, then equilibrated with approximately 100 mL of 10 mM Tris-HCl (pH 8.0, T3253, Sigma, Germany) solution. The column was further equilibrated with approximately 50 mL of 10 mM Tris-HCl (pH 8.0) solution containing 0.5 M NaCl (A501218-0001, Sangon Biotch, Shanghai, China). The sample containing the target protein was diluted and loaded onto the column. After loading, the column was washed with 10 mM Tris-HCl (pH 8.0) solution containing 0.5 M NaCl. The protein was eluted using 10 mM Tris-HCl (pH 8.0) solutions containing 15 mM, 60 mM, and 300 mM imidazole (with 0.5 M NaCl). The protein peaks were collected, and purification efficiency was analyzed by 12% SDS-PAGE electrophoresis. The protein was quantified using the BCA Protein Quantification Kit (P0010, Beyotime).

### Establishment of indirect ELISA method and serum detection

The indirect enzyme linked immunosorbent assay (iELISA) method was established as follows: The purified protein was first diluted in carbonate buffer solution (CBS, pH=9.6) to a concentration of 10 µg/mL, and 100 µL per well was added to a 96-well microplate (Corning, USA). The plate was incubated overnight at 4°C. After washing three times with PBST, 300 µL of blocking solution (5% skim milk in PBS) was added to each well and incubated at 37°C for 2 hours. The plate was washed again with PBST, then human serum diluted in PBS (1:200) was added and incubated at 37°C for 1 hour. After three more washes with PBST, 100 µL of HRP-conjugated rabbit anti-human IgG (diluted 1:10,000, A18903, Thermo Fisher, USA) was added to each well and incubated at 37°C for 1 hour. The plate was washed three times with PBST, tetramethylbenzidine (TMB, T2573, TCI, Japan) substrate solution was added, and the plate was incubated in the dark for 10 minutes for color development. The reaction was stopped with 2M H_2_SO_4_, and the OD_450_ was measured using a microplate reader (Versa Max microplate reader, MD, USA). Laboratory-stored lipopolysaccharide (LPS, provided by the China Animal Health and Epidemiology Center, 3 mg/mL) and Rose Bengal Ag (diluted 1:400, IDEXX Pourquier, Montpellier, France) were used as control antigens, and serum samples were tested in triplicate using the same procedure. Sensitivity, specificity, area under the curve (AUC), and the cut-off value were determined by receiver operating characteristic curve (ROC) analysis.

### Evaluation of cross-reactivity in indirect ELISA method

Following the procedure described above, sera from febrile patients without brucellosis were tested using the constructed *Brucella* T4SS recombinant proteins to evaluate the cross-reactivity by comparing with LPS and Rose Bengal Ag. Cross-reactivity was assessed based on the cut-off value determined by the ROC curve.

### Statistical methods

Dot plot and ROC curve analyses were conducted using GraphPad Prism version 6.05. Statistical analyses were performed using unpaired Student’s t-test and ANOVA, with a significance level set at *P* < 0.05.

## Results

### Selection of recombinant type IV secretion system proteins

Through TMT quantitative analysis, a total of 152 highly expressed proteins were identified in the wild-type strain, and 102 highly expressed proteins were identified in the vaccine strain (**Supplementary File 2**). Among the highly expressed proteins of the wild strain, we identified seven T4SS proteins, including six VirB proteins (VirB3, VirB4, VirB8, VirB9, VirB10, VirB11) and one T4SS putative outer membrane lipoprotein (BMEII0036). After predicting and removing transmembrane regions, signal peptides, and hydrophobic regions through the UniProt website (https://www.uniprot.org/uniprotkb), we constructed recombinant sequences of the seven proteins for prokaryotic expression (**Table 1**).

**Table 1:** Proteomics-based analysis and screening of Type IV secretion system proteins.

Accession	Protein	Score Sequest High Throughput (HT)	Unique Peptides	Molecular Weight (MW) [kDa]	-10 log	Location of expressed proteins (Start-end position)
Q9RPY2	VirB3	10.18	1	13.1	117.62	1-22aa, 66-116aa
Q9RPY1	VirB4	142.92	9	94.5	-∞	1-400aa
Q2YJ78	VirB8	49.17	4	26.4	-∞	68-239aa
Q9RPX6	VirB9	104.46	4	31.9	57.08	20-289aa
P0C531	VirB10	63.44	4	41.2	129.55	54-377aa
Q8FXK7	VirB11	89.57	4	40.7	-∞	1-308aa
Q8YDY8	BMEII0036	41.39	3	19	132.23	16-172aa

### Preparation of recombinant T4SS proteins

**Figure 1:**
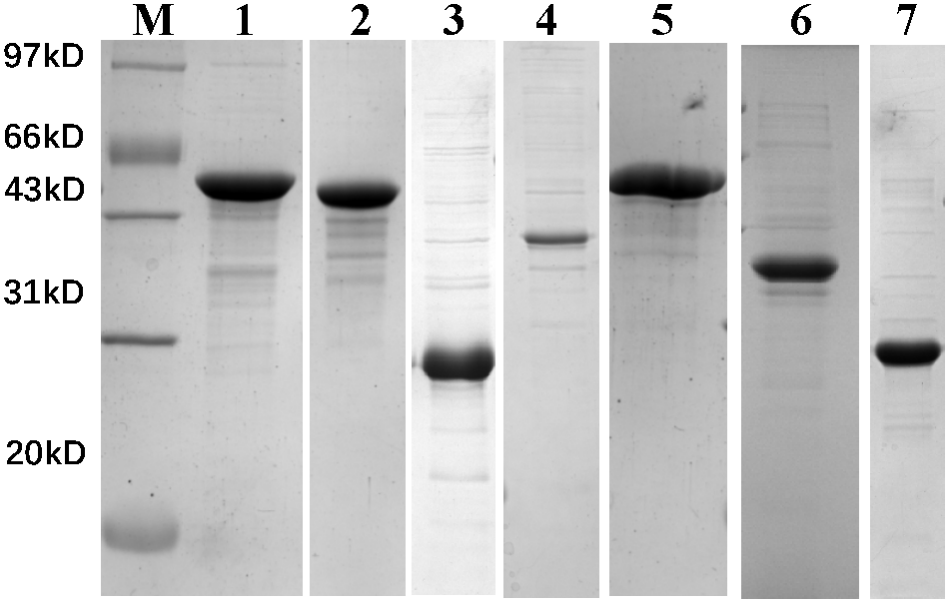
12% SDS-PAGE analysis of recombinant protein prokaryotic expression results. M, marker; Lane 1, VirB4; Lane 2, VirB11; Lane 3, VirB8; Lane 4, VirB3; Lane 5, VirB10; Lane 6, VirB9; Lane 7, BMEII0036.

All seven recombinant proteins were successfully expressed and purified through prokaryotic expression, as shown in **Figure 1** and **Supplementary File 3**. After quantification by BCA assay, the concentration was adjusted to 0.5 mg/mL in PBS and stored at -20°C for future use.

### Results of iELISA

**Figure 2:**
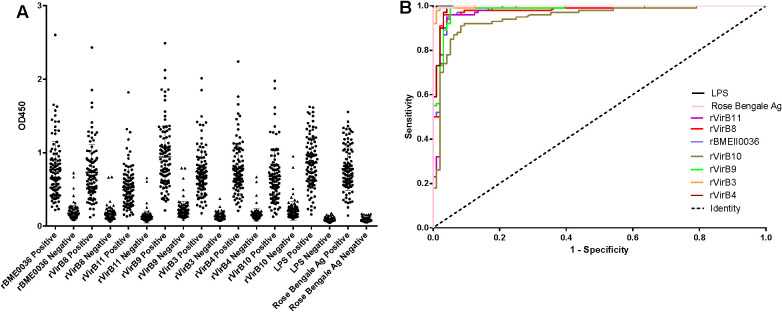
I-ELISA analysis of human serum samples. **(A)** Dotplot of the human sera. **(B)** ROC analysis of human sera.

According to the ROC curve analysis, the diagnostic accuracy of the recombinant proteins, ranked from highest to lowest, is as follows: rVirB3, rVirB4, rVirB9, rBMEII0036, rVirB8, rVirB11, and rVirB10. The area under the ROC curve (AUC) for each protein is 0.9979, 0.9914, 0.9825, 0.9817, 0.9782, 0.9764, and 0.9476, respectively, which is slightly lower compared to LPS and Rose Bengal Ag. According to the Youden index calculation, the sensitivity of these proteins is all above 0.9100, and the specificity is all above 0.9167. The highest sensitivity is 0.9900 (95% CI, 0.9455 - 0.9997) for rVirB4 and rVirB9, and the highest specificity is 0.9896 (95% CI, 0.9433 - 0.9997) for rVirB3. The sensitivity and specificity are slightly lower than those of LPS and Rose Bengal Ag. The results are presented in **Table 2**, **Figure 2**, and **Supplementary File 1**.

**Table 2:** Evaluation of ELISA results of the recombinant proteins.

Antigen	AUC	Cut-off value	Sensitivity	Specificity	Positive		Negative		Accuracy (%)	PPV (%)	NPV
			(95%CI)	(95%CI)	TP	FN	TN	FP			(%)
rVirB3	0.9979 (0.9947 to 1.001)	>0.2671	0.9800 (0.9296 to 0.9976)	0.9896 (0.9433 to 0.9997)	98	2	95	1	98.47	98.99	97.94
rVirB4	0.9914 (0.9808 to 1.002)	>0.2421	0.9900 (0.9455 to 0.9997)	0.9583 (0.8967 to 0.9885)	99	1	92	4	97.45	96.12	98.92
rVirB9	0.9825 (0.9663 to 0.9987)	>0.3348	0.9900 (0.9455 to 0.9997)	0.9479 (0.8826 to 0.9829)	99	1	91	5	96.94	95.19	98.91
rVirB8	0.9782 (0.9580 to 0.9984)	>0.2912	0.9600 (0.9007 to 0.9890)	0.9688 (0.9114 to 0.9935)	96	4	93	3	96.43	96.97	95.88
rVirB11	0.9764 (0.9541 to 0.9986)	>0.1860	0.9600 (0.9007 to 0.9890)	0.9479 (0.8826 to 0.9829)	96	4	91	5	95.41	95.05	95.79
rVirB10	0.9476 (0.9151 to 0.9801)	>0.3101	0.9100 (0.8360 to 0.9580)	0.9167 (0.8424 to 0.9633)	91	9	88	8	91.33	91.92	90.72
rBMEII0036	0.9817 (0.9653 to 0.9980)	>0.3135	0.9600 (0.9007 to 0.9890)	0.9479 (0.8826 to 0.9829)	96	4	91	5	95.41	95.05	95.79
Rose Bengale Ag	0.9994 (0.9980 to 1.001)	>0.2117	0.9900 (0.9455 to 0.9997)	1.000 (0.9623 to 1.000)	99	1	96	0	99.49	100.0	98.97
LPS	0.9999 (0.9995 to 1.000)	>0.1953	0.9900 (0.9455 to 0.9997)	1.000 (0.9623 to 1.000)	99	1	96	0	99.49	100.0	98.97

TP, true positives; TN, true negatives; FP, false positives; FN, false negatives; Accuracy, (TP+TN/TP+FN+TN+FP) ×100; PPV, positive predictive value (TP/TP+FP) ×100; NPV, negative predictive value (TN/TN+FN) ×100.

### Cross-reactivity assessment

Using iELISA and based on the determined cut-off values, cross-reactivity was observed in 2, 5, 8, 2, 1, 5, and 0 out of 40 serum samples from clinical febrile patients without brucellosis when tested with rVirB3, rVirB4, rVirB9, rBMEII0036, rVirB8, rVirB11, and rVirB10, respectively. In contrast, cross-reactivity with LPS and Rose Bengale Ag was observed in 16 and 18 cases, respectively. The cross-reactive pathogens with LPS and Rose Bengale Ag were primarily concentrated in *Escherichia coli*. Specifically, cross-reactivity with LPS included 8 cases of *Escherichia coli* infection, 3 cases of *Staphylococcus aureus*, and 1 case each of *Enterococcus faecium*, *Klebsiella pneumoniae*, *Moraxella osloensis*, *Pseudomonas putida*, and *Streptococcus dysgalactiae*. Cross-reactivity with Rose Bengale Ag was observed in 18 cases, including 7 cases of *Escherichia coli* infection, 2 cases each of *Enterococcus faecium*, *Klebsiella pneumoniae*, and *Staphylococcus aureus*, and 1 case each of *Aeromonas sobria*, *Moraxella osloensis*, *Pseudomonas aeruginosa*, *Pseudomonas putida*, and *Rothia mucilaginosa*. The results are shown in **Supplementary File 1**.

## Discussion

The T4SS is a crucial virulence factor of *Brucella*, composed of 12 protein complexes named VirB1 to VirB12, encoded by the VirB regions (Ke et al., 2015). Numerous studies have explored the potential of VirB proteins for vaccine development and serological diagnosis. For instance, Zai et al (2021) evaluated the use of VirB8 against pathogenic *Brucella* species through composite reverse vaccinology. They found that VirB8 could induce specific humoral and cellular immune responses, reduce the bacterial load of *B. abortus* S19 in mice, and provide varying degrees of protection. Yin et al (2023) used immunoinformatics to identify antigenic epitopes of VirB8 and VirB10 from the *Brucella* T4SS, screening two cytotoxic T lymphocyte epitopes, nine helper T lymphocyte epitopes, six linear B cell epitopes, and six conformational B cell epitopes for constructing a multi-epitope vaccine. Several studies have also confirmed that combining VirB10 with other proteins to create recombinant vaccines can successfully induce immune responses (Tarrahimofrad et al., 2022; Rahimnahal et al., 2023). Research has shown that VirB7 and VirB9 can induce Th1 responses in mice and dogs (Pollak et al., 2015). Additionally, there is evidence supporting the potential value of VirB5, VirB10, and VirB12 for serological diagnosis of brucellosis (Tan et al., 2012; Mirkalantari et al., 2017; Pathak et al., 2018). However, existing studies have mainly focused on individual VirB proteins, and there is a lack of systematic analysis on the use of VirB proteins for serological diagnosis of brucellosis.

In this study, we used TMT proteomics technology to identify highly expressed VirB proteins from wild-type *Brucella* strains and successfully prepared various recombinant VirB proteins for serological diagnosis. The results demonstrated that several VirB proteins (e.g., rVirB3, rVirB4, rVirB9) exhibited high sensitivity and specificity in diagnosing brucellosis. Although their performance was slightly lower than that of traditional LPS and Rose Bengale Ag, their potential as novel diagnostic antigens cannot be overlooked, the proteins still show good results for the diagnosis of human brucellosis. Besides VirB proteins, we also identified a T4SS-related protein through proteomics, namely T4SS putative outer membrane lipoprotein BMEII0036, which also showed high sensitivity and specificity when used in brucellosis diagnosis. As key components of the *Brucella* T4SS, VirB proteins not only play a role in the pathogen’s virulence mechanisms but also in its interaction with host cells, making them valuable diagnostic antigens that can more directly reflect the infection status of *Brucella*, with significant clinical application potential (Xiong et al., 2021).

In this study, we observed some differences in diagnostic performance among the various VirB proteins. For example, rVirB3 showed the best specificity, while rVirB4 and rVirB9 had the highest sensitivity. These differences might be attributed to the specific roles and expression levels of different VirB proteins during the *Brucella* lifecycle. Additionally, the antigenicity of these proteins could be influenced by factors such as amino acid sequences, spatial conformation, and glycosylation modifications (Pavlenko et al., 1990; Rahman et al., 2016; Shi et al., 2024). Therefore, future research should further explore the antigenic epitopes of these proteins and how to optimize their structures to enhance diagnostic performance.

Cross-reactivity is an important factor in assessing the specificity of diagnostic antigens. This study found that although all VirB proteins exhibited some degree of cross-reactivity, the frequency and intensity of cross-reactivity were lower than those of LPS and Rose Bengale Ag. Notably, rVirB10 showed no cross-reactivity in 40 serum samples from febrile patients without brucellosis, indicating very high specificity. This suggests that VirB proteins may have an advantage in reducing cross-reactivity when used as diagnostic antigens. However, it is important to note that cross-reactivity should be assessed with a broader and more diverse sample set to comprehensively evaluate their specificity in practical applications.

Although the TMT proteomics results indicate that other VirB proteins, including VirB1, VirB2, and VirB5-VirB7, did not exhibit any significant differences between vaccine strain *Brucella abortus* A19 and the wild-type *Brucella abortus* DT21, it is essential to further investigate the potential diagnostic value of these proteins for human brucellosis in future studies. This study provides compelling evidence that T4SS proteins play a crucial role in human brucellosis infection and offers important experimental evidence and theoretical foundation for the development of new diagnostic antigens for brucellosis. However, further research and exploration are necessary to achieve widespread clinical application of VirB proteins. In summary, VirB proteins, as key components of the *Brucella* T4SS, show great potential in the serological diagnosis of brucellosis. Future research should continue to explore their antigenicity and diagnostic performance to develop more efficient and accurate diagnostic methods for brucellosis.

## Data Availability

The original contributions presented in the study are included in the article/**Supplementary Material**. Further inquiries can be directed to the corresponding authors.

## Supplementary material

Supplementary File 1 Raw data for iELISA and information of febrile patients infected with other pathogens.

Supplementary File 2 Information on highly expressed proteins of wild strains and vaccine strains.

Supplementary File 3 Original SDS-PAGE image of recombinant proteins.
