# Supplementary material for: Preparation and evaluation of Brucella T4SS recombinant proteins in serodiagnosis of human brucellosis based on TMT-based proteomics technology
Source: Front Cell Infect Microbiol. 2025 Jan 16;14:1514046. doi: 10.3389/fcimb.2024.1514046 (PMC11779724; doi:10.3389/fcimb.2024.1514046)
Supplement: Supplementary File 3 — Original SDS-PAGE image of recombinant proteins. [file Table3.docx]

**Preparation and evaluation of *Brucella* T4SS recombinant proteins in serodiagnosis of human brucellosis based on TMT-based proteomics technology**

**Qi Wu^1^**†**, Chen Sun^2^**†**, Liping Guo^1^, Yujia Xie^1^, Jinpeng Zhang^1,3^*, Dehui Yin^1,3^***

1. Jiangsu Engineering Research Center of Biological Data Mining and Healthcare Transformation, Xuzhou Medical University, Xuzhou, Jiangsu, 221004, China

2. Department of Clinical Laboratory, Huai'an hospital of Huai'an City, Huai'an, 223200, China

3. Key Laboratory of Human Genetics and Environmental Medicine, Xuzhou Medical University, Xuzhou, 221004, China

† These authors contributed equally to this work

* Corresponding author:

**Jinpeng Zhang,** School of Public Health, Xuzhou Medical University, Xuzhou, 221004, China. E-mail: xiaopangpeng@126.com

**Dehui Yin,** School of Public Health, Xuzhou Medical University, No. 209 Tongshan Road, Xuzhou, 221004, China. E-mail: yindh16@xzhmu.edu.cn

1 M 2 3 M 4 5 6


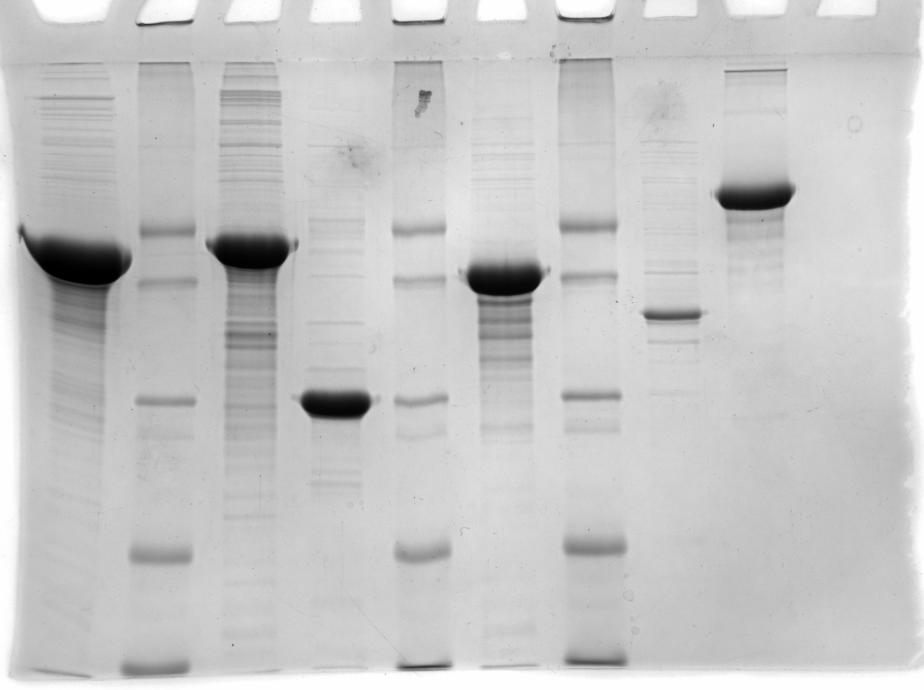


Figure S1. Original SDS-PAGE image of rBMEII0036 and rVirB3 protein. M, marker; Lane 3, rBMEII0036; Lane 6, rVirB3.

M 1 M 2 M 3


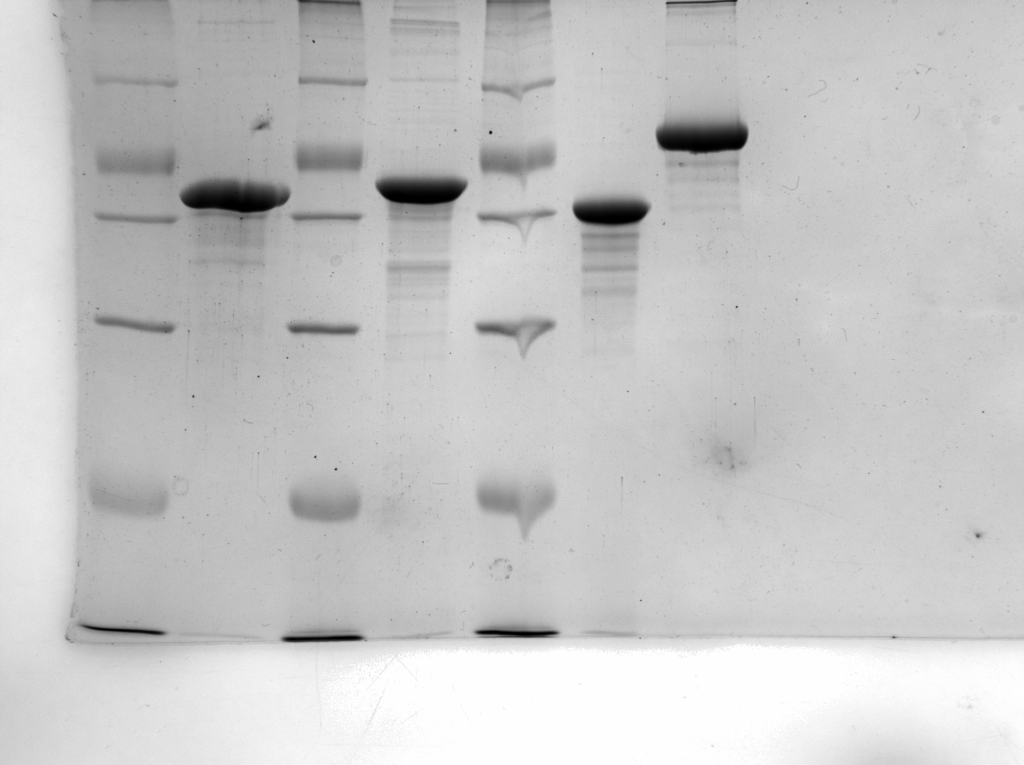


Figure S2. Original SDS-PAGE image of rVirB10, rVirB4 and rVirB11 protein. M, marker; Lane 1, rVirB10; Lane 2, rVirB4; Lane 3, rVirB11

M 1 2 3 4


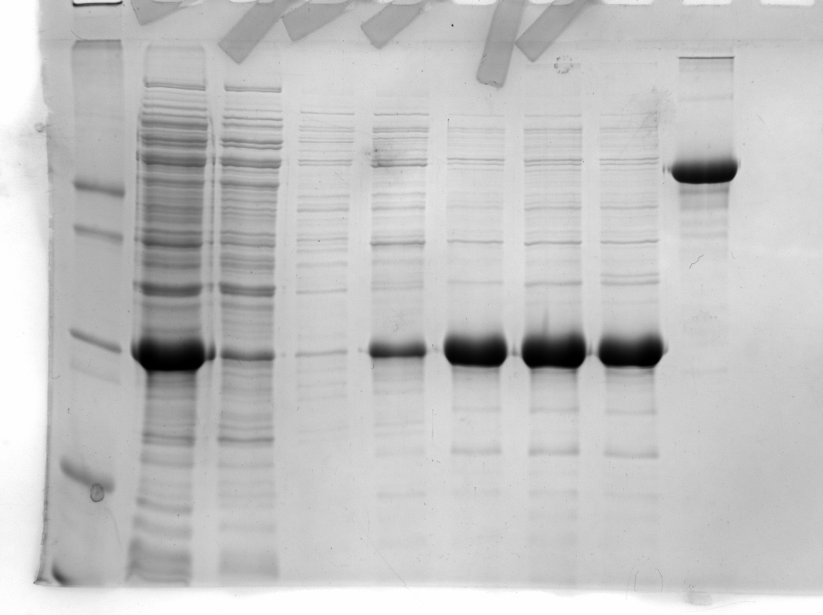


Figure S3. Original SDS-PAGE image of VirB8 protein. M, marker; Lane 1-4, rVirB8.

M 1


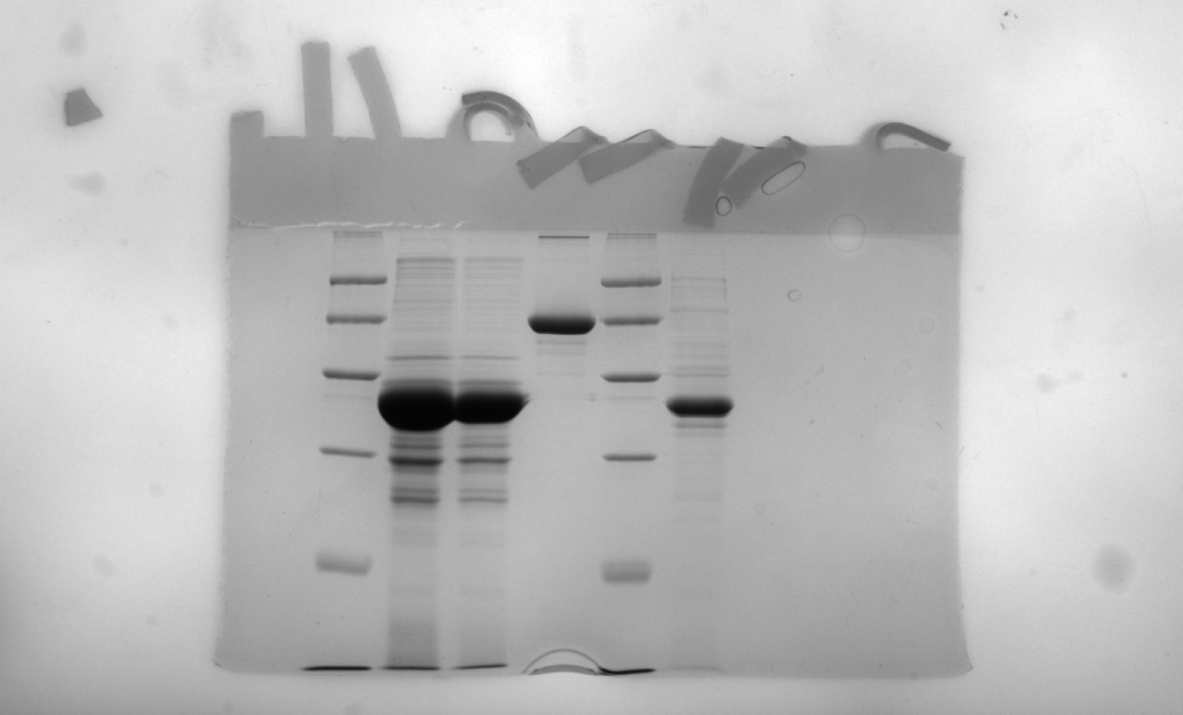


Figure S4. Original SDS-PAGE image of rVirB9 protein. M, marker; Lane 1, rVirB9.

**Additional details on TMT proteomics**

**1. Mass spectrometry detecting**

Desalted peptide mixture were loaded onto a Acclaim PePmap C18-reversed phase column（75μm×2cm, 3μm, 100 Ǻ thermo scientific) and separated with reversed phase C18 column (75μm×10cm, 5μm, 300 Ǻ, Agela Technologies) mounted on a Dionex ultimate 3000 nano LC system . Peptides were eluted using a gradient of 5–80% (v/v) acetonitrile in 0.1% formic acid over 45 min at a flow rate of 300 nL min-1 combined with a Q Exactive mass spectrometer (Thermo Fisher Scientific, MA, USA).

The eluates were directly entered Q－Exactive MS (Thermo Fisher Scientific, Waltham, MA, USA), setting in positive ion mode and data-dependent manner with full MS scan from 350-2000 m/z, full scan resolution at 70,000, MS/MS scan resoltion at 35,000.MS/MS scan with minimum signal threshold 1E+5, isolation width at 2 Da. To evaluate the performance of this mass spectrometry on the iTRAQ labeled samples, two MS/MS acquisition modes, higher collision energy dissociation (HCD) was employed. And to optimize the MS/MS acquisition efficiency of HCD, normalized collision energy (NCE) was systemically examined 30, steped 20%.

**Table S1. TMT labelled samples information**

| TMT labelling reagents | Samples |
| --- | --- |
| 126 | A19-1 |
| 129_N | A19-2 |
| 129_C | A19-3 |
| 130_N | DT21-1 |
| 130_C | DT21-2 |
| 131 | DT21-3 |

**2. Analysis of mass spectrometry results**

(1) The pre-separated components, respectively, were tested on the machine. Peptide signals were detected using a Q-Exactive mass spectrometer. After the mass spectrometry scanning is completed, the mass spectrometry raw file is obtained, and the mass spectrometry raw file is input into the PD (Proteome Discoverer 2.4, thermo) software, which will screen the mass spectrometry spectrum. The screening parameters are shown in Table S2.

**Table S2. Mass Spectrometry Screening Parameters**

| Parameters | Experimental Options |
| --- | --- |
| Parent ion mass range | 350-6000Da |
| Minimum number of peaks in secondary mass spectra | 10 |
| Signal-to-noise ratio S/N domain value | 1.5 |

(2) PD extracted spectra were searched with mascot, and after the search was finished, the PD software performed quantitative analysis based on the mascot search results and the screened spectra from the first step. The search and quantitative parameters are shown in Table S3 and Table S4.

**Table S3. Identification of search parameters**

| Parameter | Experimental Options |
| --- | --- |
| PD version | 2.4 |
| Fixed modification | Carbamidomethyl （C） |
| Variable modification | Oxidation（M），Gln→Pyro- Glu (N-term Q)，  TMT 6 plex（K），TMT 6plex（N-term） |
| Peptide tol. | 15ppm |
| MS/MS tol | 20mmu |
| Max missed cleavages | 1 |
| Enzyme | Trypsin |
| Database | uniport_bacteria  Number of sequences：335208 |

**Table S4. Quantitative Parameters**

| Parameter | Experimental Options |
| --- | --- |
| Protein ratio type^a^ | Median |
| Minimum peptides^b^ | 1 |
| Normalisation method^c^ | Median |

a: the method of peptide taking used for quantification, median refers to the method of taking the middle position; b: the minimum number of UNIQUE peptides used for quantification; c: Normalisation method, median means the median value of all quantifiable proteins in a set of samples is selected for normalisation.

(3) The results were assessed for significance of differences using ANOVA analysis of variance. It is recommended that proteins with a p-value less than 0.05, a ratio ≥ 1.2 or a ratio ≤ 0.83 be selected as differential proteins.
